# The environmental drivers of bacterial meningitis epidemics in the Democratic Republic of Congo, central Africa

**DOI:** 10.1371/journal.pntd.0008634

**Published:** 2020-10-07

**Authors:** Serge Mazamay, Hélène Broutin, Didier Bompangue, Jean-Jacques Muyembe, Jean-François Guégan

**Affiliations:** 1 Département de Microbiologie, Faculté de Médecine, Université de Kinshasa, Kinshasa, Democratic Republic of Congo; 2 MIVEGEC, IRD, CNRS, Université de Montpellier, Montpellier, France; 3 Département de Parasitologie-Mycologie, Faculté de Médecine, Université Cheikh Anta Diop (UCAD), Dakar, Sénégal; 4 Centre de Recherche en Evolution et Ecologie de la Santé (CREES), Montpellier, France; 5 Chrono-Environnement, UMR CNRS 6249 Université de Franche-Comté, Besançon, France; 6 ASTRE, INRAE, Cirad, Université de Montpellier, Campus International de Baillarguet, Montpellier, France; 7 oneHEALTH Global Research Programme, FutureEarth programme, Paris, France; University of Washington, UNITED STATES

## Abstract

**Introduction:**

Bacterial meningitis still constitutes an important threat in Africa. In the meningitis belt, a clear seasonal pattern in the incidence of meningococcal disease during the dry season has been previously correlated with several environmental parameters like dust and sand particles as well as the Harmattan winds. In parallel, the evidence of seasonality in meningitis dynamics and its environmental variables remain poorly studied outside the meningitis belt. This study explores several environmental factors associated with meningitis cases in the Democratic Republic of Congo (DRC), central Africa, outside the meningitis belt area.

**Methods:**

Non-parametric Kruskal-Wallis’ tests were used to establish the difference between the different health zones, climate and vegetation types in relation to both the number of cases and attack rates for the period 2000–2018. The relationships between the number of meningitis cases for the different health zones and environmental and socio-economical parameters collected were modeled using different generalized linear (GLMs) and generalized linear mixed models (GLMMs), and different error structure in the different models, i.e., Poisson, binomial negative, zero-inflated binomial negative and more elaborated multi-hierarchical zero-inflated binomial negative models, with randomization of certain parameters or factors (health zones, vegetation and climate types). Comparing the different statistical models, the model with the smallest Akaike’s information criterion (AIC) were selected as the best ones. 515 different health zones from 26 distinct provinces were considered for the construction of the different GLM and GLMM models.

**Results:**

Non-parametric bivariate statistics showed that there were more meningitis cases in urban health zones than in rural conditions (*χ*^*2*^ = 6.910, *p*-value = 0.009), in areas dominated by savannah landscape than in areas with dense forest or forest in mountainous areas (*χ*^*2*^ = 15.185, *p*-value = 0.001), and with no significant difference between climate types (*χ*^*2*^ = 1.211, *p*-value = 0,449). Additionally, no significant difference was observed for attack rate between the two types of heath zones (*χ*^*2*^ = 0.982, *p*-value = 0.322). Conversely, strong differences in attack rate values were obtained for vegetation types (*χ*^*2*^ = 13.627, *p*-value = 0,001) and climate types (*χ*^*2*^ = 13.627, *p*-value = 0,001). This work demonstrates that, all other parameters kept constant, an urban health zone located at high latitude and longitude eastwards, located at low-altitude like in valley ecosystems predominantly covered by savannah biome, with a humid tropical climate are at higher risk for the development of meningitis. In addition, the regions with mean range temperature and a population with a low index of economic well-being (IEW) constitute the perfect conditions for the development of meningitis in DRC.

**Conclusion:**

In a context of global environmental change, particularly climate change, our findings tend to show that an interplay of different environmental and socio-economic drivers are important to consider in the epidemiology of bacterial meningitis epidemics in DRC. This information is important to help improving meningitis control strategies in a large country located outside of the so-called meningitis belt.

## Author summary

Bacterial meningitis remains an international public health threat. The most affected area worldwide is the Sahelian region between Senegal and Ethiopia called the meningitis belt. In parallel recurrent epidemics have also occurred out of the belt in Africa for the last twenty years. While environmental, socioeconomic and demographic factors are well described to explain the epidemiology of meningitis in the belt, very few studies have focused on the role of these factors to understand meningitis epidemics outside of the belt. Based on epidemiological, socio-economic, demographic and environmental data collected for the period 2000–2018 in the Democratic Republic of Congo (DRC), we used different statistical methods to explore the links between meningitis cases and the different explored factors. Our results showed that urbanized areas where populations have a low economic index of well-being, high latitude and longitude eastwards, medium to low altitude level, savannah-type vegetation, medium temperature value, are risk factors of meningitis outbreaks from North to South in the DRC. This information is important to help improving meningitis control strategies in a large country located outside the so-called meningitis belt.

## Introduction

Global burden of bacterial meningitis was estimated to 5 million of new cases and 290,000 deaths globally in 2017 [1], mainly due to three major bacteria: *Neisseria meningitidis* (Nm), *Streptococcus pneumoniae* (Sp) and *Haemophilus influenzae* type b (Hib) [2]. Bacterial meningitis remains a major public health in Africa, and in particular in the Sub-Saharan region stretching from Senegal to Ethiopia, called the « Meningitis Belt » [3, 4].

In the African meningitis belt, bacterial meningitis incidence exhibits a seasonal trend with peaks during the dry season which then subsides with the onset of the rainy season [5, 6].

The massive introduction of the affordable conjugate vaccine against meningococcal serogroup A started in December 2010, the MenAfriVac [7, 8] in addition to the recent introduction of the 10–13 pneumococcal serotypes (from 2013) vaccines in infants through the Expanded Programme of Immunisation (EPI) in many countries in the meningitis belt [9], induced a strong decrease of meningitis incidence in this part of the world. However, bacterial meningitis remains a public health threat in the African continent mainly due to the persistence of other meningococcal *Neisseria* serogroups principally Nm C, Nm W, to *Haemophilus influenzae* (Hi) infection and high incidence of pneumococcal (Sp) meningitis in adults. This is strongly observed in the meningitis belt, while the ecology of meningitis disease in Africa but outside the belt is still poorly understood.

A better understanding of the mechanisms and factors which intervene behind this epidemiology is therefore needed, along with appropriate statistical models allowing the identification of environmental, demographic and socio-economic factors of meningitis epidemics not only in the meningitis belt but also outside this belt. This is highly relevant for better preventive strategies against this infection and the adjustment of disease surveillance. Both the epidemiological and ecological landscapes represent appropriate tools to merge for analyzing the occurrence and geographical distribution of infectious diseases and their interactions with environmental parameters in both space and time [10, 11].

Many studies at different spatial scales and in different countries of the meningitis belt have been carried out, highlighting the role of dust, relative humidity and Harmattan winds in the different phases of meningitis epidemics [5, 12, 13, 14]. The link between climate-environment and meningitis is becoming more and more evident through many scientific works and some environmental factors or proxies are also considered in early warning systems and prevention [5, 15, 16]. Through the countries of the meningitis belt, previous modeling efforts relied on a wide range of unknown parameter values [17] given the lack of surveillance data from which parameters could be estimated. Others have used incidence data for model fitting at low spatial resolution, mainly data aggregated at district level [18, 19]. This does not allow for the differentiation between dry seasons with localized epidemics and dry seasons without localized epidemics, as localized epidemics usually can be seen at the health center level only [20, 21]. Other studies are underway in the meningitis belt in order to highlight other drivers that may modify the epidemiology of this deadly and debilitating disease. However, the upsurge of meningitis epidemic outbreaks outside the Sahelo-Sudanian belt, which is at the origin of the Southern meningitis epidemics’ hypothesis [22, 23], is still of little interest and remains poorly studied despite the negative impact of this disease from a public health point of view in those regions. The evidence of meningitis seasonality and environmental, demographic and socio-economic variables that can influence the incidence of disease cases remains also poorly studied.

This article sought to evaluate the effect of environmental, demographic and socio-economic drivers on meningitis diseases risk in the Democratic Republic of Congo (DRC) where bacterial epidemics are recurrent and present a high diversity in meningitis incidences.

## Methods

### Study area

The DRC is an African country crossed by the Equator line, located at the 5° North latitude and 13° South Latitude, with a 2,345,409 km^2^ surface area. The population size is estimated at 85,026 million inhabitants, a low density of 21 inhabitants per km^2^, and with about 50% of under 15 years-old, and 55–60% of the total population living in rural areas. The country shares limits with 9 distinct countries (to the north by the Central African Republic and South Sudan, to the west by the Republic of Congo, to the east by Uganda, Rwanda, Burundi and Tanzania, to the south-east, by Zambia and in the south-west by Angola).

DRC presents diverse geography including a basin in the center of the country (48% of the total surface), with a mean altitude of 350 m and covered by dense tropical and equatorial forests. Uplands surround this part of the country up to the borders except in the eastern part where uplands end with mountains with a mean altitude higher than 1000 m. This geographic mosaic also includes 4 different climates across the country: an equatorial climate in the center of the country (the basin or pool), a tropical and humid climate in the north and south, a temperate climate in the east and a mountain climate in the extreme east; and this conditions the existence of different biomes made of dense forest, moist forest, dry forest, meadow, savannah, bush in the north and south and mountain forests in the east [24].

Additionally to the dense tropical forest in the basin, the important river network (i.e., the Congo river of 4700 km and its numerous tributaries and lakes) constitutes an important geographic characteristic of the country. The socio-economic situation of the population is very poor, especially in rural areas where populations live from traditional agriculture, fishing and bushmeat hunting.

The health system is organized into 3 administrative levels, i.e., central, intermediary and peripheric. As of 2015, the country is divided into 26 sanitary provinces [25], 515 health zones (a geographical entity of an average of 6000 to 10,000 km^2^ including a population of at least 100,000 inhabitants) and 8504 health areas (a geographical entity of an average of 300 to 500km^2^ including a population of at least 10,000 inhabitants) [26].

In connection with the global fight against meningitis, a standard definition of bacterial meningitis suspected case and epidemic disclosed by the General Directorate for the fight against the disease (DGLM), is used through the various health structures (General Hospitals, health centers and others) of the 515 health zones [27]. A suspected case of acute meningitis is a patient with a sudden onset of fever (>38.5°C rectal or 38.0°C axillary) with stiff neck. In patients under 1 year of age, a suspected case of bacterial meningitis occurs when fever is accompanied by a bulging fontanelle.

In the DRC the epidemic threshold is defined by 15 cases per 100,000 inhabitants per week for one week in a district with a population between 30,000 and 100,000 inhabitants, or by 5 cases in one week or doubling of cases during 3 weeks for a population of less than 30,000 inhabitants. However, when the epidemic risk is low (for example, no epidemic for 3 years or warning threshold exceeded at the beginning of the dry season), the recommended epidemic threshold is 10 cases per 100,000 people per week [27].

#### Epidemiological data

We collected epidemiological data from the weekly numbers of suspected cases and deaths reported in the DRC national Integrated Diseases Surveillance System (IDSR) collected by the national health system from the Ministry of Health, between 2000 and 2018 (S1 Data), at the health zone area scale. Meningitis suspected cases were defined according to the WHO guidelines [27].

The cases were notified to the hierarchic level by the health structures (hospital and health centers) in the 515 different health zones and from sentinel health zone sites, attached to health areas in the provinces of Haut Katanga (Lubumbashi) and the city-province of Kinshasa (Lingwala, Kalembelembe and Kingasani).

Patients with suspected meningitis who were evaluated at reference centers or at sentinel sites structures had a lumbar puncture performed to collect cerebrospinal (CSF) fluid for laboratory testing by Gram stain and culture or latex agglutination, when available, or by Polymerase Chain Reaction (PCR) at the Institut National de Recherche Biomédicale (INRB), Kinshasa. Between 2008 to 2012, patients were primarily infected by *S*. *pneumoniae* (50%), *N*. *meningitidis* (13%), *H*. *influenzae* (2%) or other pathogens (35%) identified by CSF culture or PCRs performed. 486 CSF samples were tested via culture (100%) and 267 via PCR (54.93%) [28].

#### Population data

Population sizes of health areas were collected from the Expanded Programme on Immunization (EPI), program of Public Health Ministry. They correspond to estimations based on the last census performed in 1984 with a calculated annual growth rate of 1.03% applied for the following years.

#### Shapefile data

Issued from WHO Healthmapper (a WHO information and mapping application for public health).

#### Socio-environmental data

**Climate data** has been downloaded from WorldClim—Global Climate Data [29]. They concern 30-years average monthly rainfall and temperature data (1970–2000). The variables are collected at the scale of 30 arc seconds (~ 1km^2^). Each zip file contains 12 GeoTiff (.tif) files, 1 per month (January = 1, December = 12). The raster image was superimposed on the DRC 515 health zones Shapefile, and we extracted the average, minimum, maximum, and mean temperature (in °C) and precipitation data (in mm) for each health zones using ArcGIS (ver 10.6.1). For all these data we used the average values.

**The altitude data** for health zones are from the Shuttle Radar Topography Mission (SRTM) in the European soil data center (ESDAC) [30]. Outside the USA and their associated territories, an altitude measurement is taken every 90 m. This makes it possible to draw quite high accuracy isolines of altitudes throughout the DRC. Information is available in the form of rasters, and backgrounds were obtained from SRTM 90m Digital Elevation Database v4.1 site h [31].

In order to cover the entire territory of the DRC, 19 raster images have been downloaded. These were subsequently mosaic into a single image. The raster image was superimposed on the 515 health zones shapefile of the DRC, and the average altitude data extraction for each health zone was performed using ArcGIS.

**The latitude and longitude data** of the health zones were extracted from the shapefile of the 515 health zones using the *R* software.

**Bushfire** data by health zone are dated and geo-localized from MODIS images provided by NASA site [32, 33].

**Livestock** rearing activity in the health zone (1 = Health zone without livestock breeding activities, 2 = HZ with small-scale livestock activities, 3 = HZ with large livestock activities).

**The Index of Economic Well-being** (IEW) was estimated during the second Demographic and Health Survey conducted (DHS) in the DRC between 2013 and 2014 [34]. The IEW is built on information on household ownership of certain durable goods (television, radio, etc.) and on certain housing characteristics (availability of electricity, type of drinking water supply, type of toilets, flooring materials, number of rooms used for sleeping, type of fuel for cooking, etc.). The index is constructed as follows: 1) assigning a weight or a score to each household property or characteristic (score or coefficient) generated from a principal component analysis; 2) standardization of scores according to a standard normal distribution of mean 0 and standard deviation of 1; 3) allocation and summation of all scores for each household; 4) ranking of households in ascending order of total score according to five equal size categories called quintiles, ranging from 1 (the lowest quintile) to 5 (the highest quintile). We used the classification of different districts according to this index found in the above national survey. We summed the lowest quintile with the second quintile to correspond to a poverty proxy that we assigned to the health zones corresponding to the district.

All of these parameters were used as independent variables in the different statistical models.

#### Statistical methods

Non-parametric Kruskal-Wallis’ tests were used to establish the difference between the different types of health zones, climate, vegetation in relation to the number of cases and attack rates for the period of study.

The relationships between the number of meningitis cases in the 515 health zones and environmental, demographic and socio-economic parameters (population size, population density, type of health zone, type of climate, type of vegetation, altitude, latitude, longitude, precipitation, minimum temperature, mean temperature, maximum temperature, large livestock existence, proportion of bushfires and index of economic well-being) were modeled using different generalized- (GLMs) and generalized linear mixed models (GLMMs), and different error structure in the different models, i.e., Poisson, binomial negative, zero-inflated binomial negative and more elaborated multi-hierarchical zero-inflated binomial negative models, with randomization of certain parameters or factors (health zones, vegetation types, climate types) [35, 36].

Comparing the different statistical models, the models with the smallest AIC (Akaike’s Information Criterion) were selected as the best ones. 515 different health zones from 26 provinces were considered for the construction of the different GLM and GLMM models.

Taking into account the data type of the parameters mentioned above in our possession, we developed GLMs and GLMMs models. In particular, GLMMs are generalized linear models with mixed effects. They are used to analyze non-numerical continuous—such as counting data, binary responses, or proportions (for the GLM portion) -, and when the data are not independent (for the mixed part) [36]. The count type data are not distributed according to a normal parametric distribution, but according to a Poisson or Poisson-like distribution. Given this distribution law, the variance of the residuals is not constant but proportional to the mean counts predicted by the model. We thus used GLMM with a logit link and a Poisson error distribution, then a binomial negative error distribution, and finally a zero-inflated binomial negative error distribution in turn. Since we generated many different statistical models in the present work, models with Poisson structure which never succeeded to converge are not illustrated in the present work.

Using these different parameters, the number of expected meningitis cases and or the attack rate is given by the formula of the following structure:

$$\begin{array}{l} Number\;of\;meningitis\;cases\;or\;mean\;attack\;rate=f(PopDensity+Popsize+Type\;of\;climate+\\ Type\;of\;vegetation+Altitude+Latitude+Longitude+mean\;Precipitation+Tempmin+Tempmax+\\ Tempmoy+BushFire+livestock+IEW,\;random=∼1|health\;zone,\;method="ML"). \end{array}$$ (Eq 1)

With:

Number of cases = number of meningitis cases expected or mean attack rate

PopDensity = Population density of the health zone and Popsize = Population size in number of inhabitants

TypeZS = Type of health zone (1 = urban; 2 = rural and urban-rural)

Typeclimate = Climate type (1 = Montagnard, 2 = Equatorial; 3 = Tropical humid)

Vegetation type = type of vegetation (1 = forest in mountain area, 2 = dense forest; 3 = savannah)

Altitude = Altitude of the health zone

Longitude = Longitude of the health zone

Precipitation = mean amount of precipitation in millimeters

Tempmin = minimal Temperature in Celsius degree

Tempmax = maximum temperature in Celsius degree

Tempmoy = Mean temperature in Celsius degree

Bushfire = bushfire existence in the health zone

Livestock = livestock rearing activity in the health zone

IEW = index of economic well-being.

In Eq 1 above, health zone was used as a random variable. In subsequent GLMMs, we also introduced the vegetation type and climate type as random variables in turn, and then complexified the models in considering the hierarchical nature of several independent variables (e.g., health zones nested within vegetation types) in the random sub-equation of Eq 1.

Since we generated many different statistical models in this work with different types of error structure, we decided to present only GLMM results and not GLM ones, to not illustrate Poisson error structure models, and to present only the best-fitted minimum models with the smallest AIC values. Only the statistically significant dependent variables, derived from the negative binomial, zero-inflated negative binomial and multi-hierarchical zero-inflated negative binomial models were then retained as the best determinants of meningitis case incidence and attack rate in the DRC. The analyses were performed with the software Stata ver. 16 (StataCorp, LLC, 2019).

## Results

Between 2000 and 2018, there were 118,378 cases reported by the Ministry of Health through the DGLM with a lethality of 11.5% across the 515 health zones. The incidence of cases is more marked in the regions located in the North and South-East of the country and in the provincial city of Kinshasa. [28].

**Fig 1:**
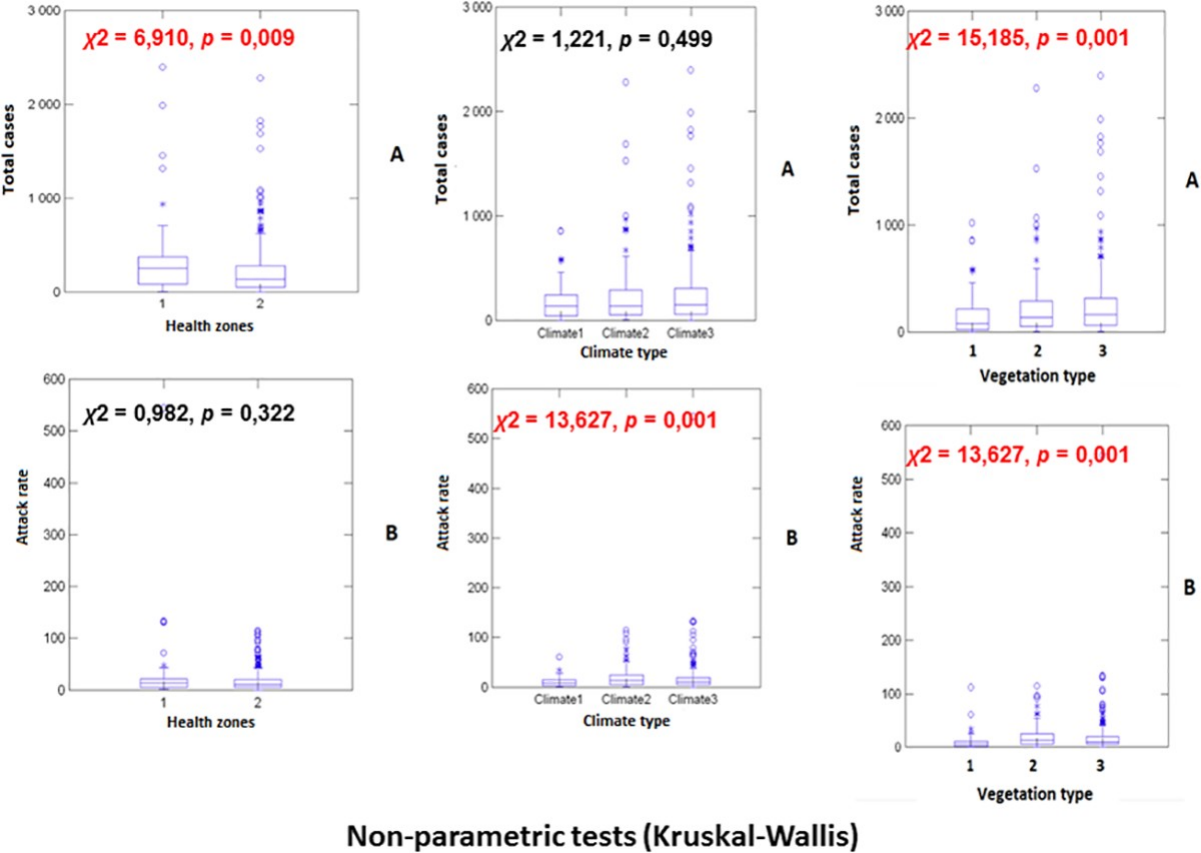
Figures issued to non-parametric test analysis (Kruskal-Wallis). File in. TIFF extension.

Non-parametric Kruskal-Wallis’ tests show that more meningitis cases occur on average in urban health zones than in rural ones (*χ*^*2*^ = 6.910, *p*-value = 0.009), with more cases observed in dominant savannah vegetation areas than in dense forests or mountainous forest zones (*χ*^*2*^ = 15.185, *p*-value = 0.001; all pairwise comparisons were also significant, *p*-value = 0.001). No difference in disease incidence was observed across the different types of climates (*χ*^*2*^ = 1.221, *p*-value = 0.499). Concerning attack rate values, no difference was observed between the two health zones, i.e., urban versus rural/urbano-rural (*χ*^*2*^ = 0.982, *p*-value = 0.322; all pairwise comparisons were also significant, *p*-value = 0.001). Important differences were obtained for climate (*χ*^*2*^ = 13.627, *p*-value = 0.001) and vegetation (*χ*^*2*^ = 13.627, *p*-value = 0.001; all pairwise comparisons were also significant, *p*-value = 0.001 except between equatorial climate and humid tropical, where *p*-value = 0.027) types (see figures in Fig 1).

From the different GLMM models we generated, the total number of meningitis cases across the different health zones in the DRC is best explained by a consistent set of explanatory environmental, demographic and socio-economic variables, and this whatever the statistical models developed and its error structure (see Table 1).

**Table 1:** Summary of generalized linear mixed models (GLMM) to explain the total number of meningitis cases across the 515 health districts in the Democratic Republic of Congo (DRC).

Error structure Determinant variables	Negative binomial			Zero-inflated negative binomial (with Population density and Population size zero-inflated)			Multi-hierarchical zero-inflated negative binomial		
	**Healthzone as random variable**						**HealthZones included in Climatetypes, as random variables**		
	**Coef.**	**(P>|z|)**	**[95% Conf. Interval]**	**Coef.**	**(P>|z|)**	**[95% Conf. Interval]**	**Coef.**	**(P>|z|)**	**[95% Conf. Interval]**
Popdensity									
Altitude	-.0006	(0.005)	[-.0009 -.0002]	-.0005	(0.013)	[-.0009 -.0001]	-.0005	(0.008)	[-.0009 -.0001]
Longitude	.0794	(0.000)	[.0531 .1057]	.0623	(0.000)	[.0344 .0902]	.0742	(0.000)	[.0471 .1012]
Latitude				.0224	(0.045)	[.0005 .0444]	.0179	(0.102)	[-.0035 .0395]
Maxtemp	-.1599	(0.008)	[-.2781 -.0419]	-.2046	(0.001)	[-.3239 -.0852]	-.1735	(0.004)	[-.2926 -.0543]
MeanTemp	.2488	(0.000)	[.1473 .3503]	.2948	(0.000)	[.1917 .3978]	.2532	(0.000)	[.1518 .3546]
IEW	-.0107	(0.000)	[-.0150 -.0064]	-.0182	(0.000)	[-.0225 -.0138]	-.0116	(0.000)	[-.0161 -.0072]
	**Climate type as random variable**						**HealthZones included in Vegetationtypes, as random variables**		
Popdensity									
Altitude	-.0006	(0.005)	[-.0009 -.0002]				-.0006	(0.005)	[-.0009 -.0002]
Longitude	.0796	(0.000)	[.0532 .1061]	.0332	(0.011)	[.0077 .0587]	.0794	(0.000)	[.0531 .1057]
Latitude				.0697	(0.000)	[.0476 .0918]			
Mintemp				-.1099	(0.006)	[-.1878 -.0319]			
Maxtemp	-.1600	(0.008)	[-.2783 -.0418]	-.2874	(0.000)	[-.4420 -.1327]	-.1599	(0.008)	[-.2781 -.0419]
MeanTemp	.2514	(0.000)	[.1488 .3541]	.4151	(0.000)	[.2266 .6036]	.2488	(0.000)	[.1473 .3503]
IEW	-.0116	(0.000)	[-.0176 -.0057]	-.0085	(0.000)	[-.0129 -.0041]	-.0107	(0.000)	[-.0151 -.0064]
	**Vegetation type as random variable**								
Altitude	-.0006	(0.005)	[-.0009–0002]						
Longitude	.0794	(0.000)	[.0531 .1057]	.0472	(0.000)	[.0212 .0731]			
Latitude				.0661	(0.000)	[.0438 .0884]			
Mintemp				-.1078	(0.006)	[-.1849 -.0307]			
Maxtemp	-.1599	(0.008)	[-.2781 -.0419]	-.2518	(0.001)	[-.4018 -.1018]			
MeanTemp	.2488	(0.000)	[.1473 .3503]	.4021	(0.000)	[.2198 .5845]			
IEW	-.0107	(0.000)	[-.0151 -.0064]	-.0106	(0.000)	[-.0149 -.0061]			

Note: **Only minimal models based on their AIC values are illustrated in this table.**

Controlling for the effect of population size and population density in the different models, latitude was positively associated with meningitis cases with higher latitudinal areas in the DRC exhibiting more meningitis cases than lower latitudinal areas. For longitude, we also observed a spatial trend of more meningitis cases in Eastern regions in the DRC than in the Western part of the country. Altitude was also associated negatively with disease incidence indicating that mountainous and hilly regions were less affected by meningitis cases than low altitude areas like valleys and river catchment areas. Maximum temperature was correlated negatively with the number of meningitis cases with upper temperatures acting probably as a limit for the development of the disease agent life-cycle. Mean temperature was positively associated to the number of disease cases with an optimal temperature range being also important for the circulation of the different disease agents causing bacterial meningitis in the region. IEW was negatively associated to disease incidence with higher values for this index of well-being favoring less cases of meningitis in the country.

We observed that minimum temperature was also retained in minimal models, acting negatively on the number of meningitis cases, which tends to indicate the same process of a lower temperature limit for the development of this disease in the DRC (Table 1). The adjusted coefficients of determination (adjusted-*R*^2^) for the different GLM and GLMM models to explain the number of total meningitis cases across health zones fluctuated between adjusted-*R*^2^ = 0.109 (*p*<0.0001, corrected-AIC = 7268.998, first-order autocorrelation = 0.065) for minimum negative binomial models, adjusted-*R*^2^ = 0.267 (*p*<0.0001, corrected-AIC = 5634.442, first-order autocorrelation = 0.035) for minimum negative binomial zero-inflated models and adjusted-*R*^2^ = 0.322 (*p*<0.0001, corrected-AIC = 4821.318, first-order autocorrelation = 0.026) for the most elaborated minimal multi-hierarchical zero-inflated binomial negative models (see also Table 1).

Concerning the attack rates, we also observed the same set of coherent environmental and socio-economic variables as previously described for meningitis incidence in the DRC (see Table 2). The adjusted-*R*^2^ values ranged from 0.152 (*p*<0.001, corrected-AIC = 4964.880, first autocorrelation = 0.019) for minimum negative binomial models, 0.196 (*p*<0.001, corrected-AIC = 3865.422, first-order autocorrelation = 0.017) for minimum negative binomial zero-inflated models and 0.256 (*p*<0.001, corrected-AIC = 3257.754, first-order autocorrelation = 0.016) for the multi-hierarchical zero-inflated negative binomial models (see Table 2).

**Table 2:** Summary of generalized linear mixed models (GLMM) to explain the mean attack rate of meningitis across the 515 health districts in the Democratic Republic of Congo (DRC).

Error structure Determinant variables	Negative binomial			Zero-inflated negative binomial (with Population density and Population size zero-inflated)			Multi-hierarchical zero-inflated negative binomial		
	**Healthzone as random variable**						**HealthZones included in Climatetypes, as random variables**		
	**Coef.**	**(P>|z|)**	**[95% Conf. Interval]**	**Coef.**	**(P>|z|)**	**[95% Conf. Interval]**	**Coef.**	**(P>|z|)**	**[95% Conf. Interval]**
Popdensity	-.00002	(0.000)	[-.00004 -.00001]						
Altitude	-.0005	(0.007)	[-.0009 -.0001]	-.0005	(0.011)	[-.0009 -.0001]	-.0005	(0.007)	[-.0009 -.0001]
Longitude	.0692	(0.000)	[.0421 .0963]	.0529	(0.000)	[.0248 .0811]	.0642	(0.000)	[.0372 .0912]
Latitude	.0264	(0.014)	[.0054 .0475]	.0318	(0.004)	[.0102 .0535]	.0279	(0.009)	[.0069 .0489]
Maxtemp	-.1815	(0.003)	[-.3013 -.0617]	-.2131	(0.001)	[-.3355 -.0907]	-.1874	(0.002)	[-.3081 -.0667]
MeanTemp	.2583	(0.000)	[.1540 .3625]	.3046	(0.000)	[.1976 .4116]	.2654	(0.000)	[.1611 .3697]
IEW	-.0117	(0.000)	[-.0174 -.0059]	-.0210	(0.000)	[-.0254 -.0167]	-.0143	(0.000)	[-.0187 -.0099]
	**Climate type as random variable**						**HealthZones included in Vegetationtypes, as random variables**		
Popdensity	-.0001	(0.017)	[-.0003–20.0000]						
Altitude	-.0005	(0.009)	[-.0009 -.0001]				-.0005	(0.007)	[-.0009 -.0001]
Longitude	.0603	(0.000)	[.0331 .0875]	.0272	(0.037)	[.0016 .0528]	.0642	(0.000)	[.0372 .0912]
Latitude	.0300	(0.005)	[.0089 .0511]	.0778	(0.000)	[.0561 .0995]	.0279	(0.009)	[.0069 .0489]
Mintemp				-.0903	(0.020)	[-.1663 -.0142]			
Maxtemp	-.1939	(0.002)	[-.3138 -.0739]	-.2757	(0.001)	[-.4313 -.1201]	-.1874	(0.002)	[-.3081 -.0667]
MeanTemp	.2819	(0.000)	[.1773 .3865]	.3933	(0.000)	[.2044 .5821]	.2654	(0.000)	[.1611 .3697]
IEW	-.0167	(0.000)	[-.0216 -.0119]	-.0112	(0.000)	[-.0156 -.0068]	-.0143	(0.000)	[-.0187 -.0099]
	**Vegetation type as random variable**								
Altitude	-.0005	(0.007)	[-.0009 -.0001]						
Longitude	.0642	(0.000)	[.0372 .0912]	.0396	(0.003)	[.0137 .0654]			
Latitude	.0279	(0.009)	[.0069 .0489]	.0767	(0.000)	[.0549 .0985]			
Mintemp				-.0869	(0.027)	[-.1638 .0101]			
Maxtemp	-.1874	(0.002)	[-.3081 -.0667]	-.2414	(0.002)	[-.3969 .0858]			
MeanTemp	.2654	(0.000)	[.1611 .3697]	.3743	(0.000)	[.1852 .5635]			
IEW	-.0143	(0.000)	[-.0187 -.0099]	-.0133	(0.000)	[-.0177 -.0089]			
				**Livestock, as random variable**					
Altitude				-.0006	(0.005)	[-.0010 -.0002]			
Longitude				.1123	(0.000)	[.0826 .1420]			
Maxtemp				-.2518	(0.000)	[-.3805 -.1232]			
MeanTemp				.4495	(0.000)	[.3361 .5629]			
IEW				-.0187	(0.000)	[-.0232 .0142]			

Note: **Only minimal models based on their AIC values are illustrated in this table.**

## Discussion

Despite the existence of vaccination and the effectiveness of antibiotic therapy, bacterial meningitis remains an important source of morbidity and mortality among populations, especially in Sub-Saharan Africa. Studies conducted in the African meningitis belt countries have shown a link between several environmental factors and the incidence of cases. These studies have focused on the meningitis belt, with few studies conducted outside this belt, such as in the DRC, where meningitis cases are officially registered with the government.

The present study conducted in the DRC, a country that reported nearly 118,378 cases of meningitis between 2000 and 2018 with a lethality of 11.5%, close to the lethality found in countries of the meningeal belt [37] aimed to understand for the first time the main environmental, demographic and socio-economic drivers of meningitis cases and attack rates over this large country of central Africa.

We previously demonstrated a spatial distribution of cases in 8 clusters at the spatial scale [28]. These meningitis clusters are distributed in health provinces with a high average attack rate, particularly in the North-East and South-East provinces and in the Kinshasa province in the south-west of the country.

This study allowed us to provide the first explanations on the spatial heterogeneities observed at the level of health zones taken into account several important environmental, demographic and socio-economic variables that could have a link with the incidence of meningitis cases.

Indeed, we showed a significant trend between the number of bacterial meningitis cases and several environmental and socio-economic factors, including: type of health zone, altitude, latitude, longitude, temperature, vegetation, type of climate and the economic well-being index across the different health zones.

The DRC constitutes a very large country harboring different climatic and biome regions. This important spatial heterogeneity in environmental conditions across the country also mirrors a wide variability in administrative, socio-economic, educational or health systems in the 515 districts surveyed during this work. All these conditions may predispose some particular areas in DRC to be more prone to the development of these bacterial infections.

We show here that a set of drivers of meningitis cases and attack rates is retained by the different statistical models. First, obviously the population size or the population density of the different health districts are positively associated with the development of meningitis outbreaks in DRC, and we took into consideration these two demographic parameters to analyze the effects of other environmental or socio-economic factors, the two demographic parameters being kept constant into analysis. Second, both latitudinal and longitudinal gradients are important in the development of meningitis in this African country. We observe, in general, more meningitis cases and severe attack rates at higher latitude and longitude eastwards in spite of the fact that Kinshasa and its different districts represent an important place for the development of meningitis outbreaks. Then, altitude constitutes another important environmental parameter in the development of meningitis in the DRC, with lower altitude areas like large plains and river catchment areas favoring the development of the bacterial disease. The role of altitude could be explained by long-distance transport of dust in upper layers of the atmosphere that may expose microbes to challenging conditions [38]. For example in the epidemiology of malaria, the incidence of cases decreases when altitude increases [39, 40]. Then higher uplands and mountains do not favor the development of meningitis probably due to the prevailing climatic conditions [41] and barriers that prevent unsurmountable barriers to human presence and migration. Indeed, temperature and notably its maximum and mean values appear as two important parameters to explain meningitis cases and attack rates. Regions in DRC with the warmest temperature values tend to see less or no epidemic outbreaks of meningitis, which represents a physiological limit for the development of the disease bacterial agent life-cycle favoring its autolysis [42]. In addition, mean temperature is important for the development of meningitis, which corresponds to a range of optimal temperature values for the circulation of the different bacterial strains causing meningitis [41]. In some statistical models, we also obtained minimum temperature as an explanatory variable, which is entirely consistent with the explanations given above of a minimal temperature limit to the expansion of bacteria. It is important to note here that precipitation was never retained in the many different statistical models we produced; this can be explained by important collinearity that may exist with other dependent variables retained by our models but also by the fact that at the scale of the country the range in rainfall values is too low to be retained in generalized linear models, that which introduced statistical consequences in the models, i.e., Elton’s sound hypothesis [43]. Livestock condition was also not retained in the present results, suggesting that areas exhibiting large herds, and so plausible flows of pastoralists, may not influence statistically the development of meningitis epidemics.

The role of the index of economic well-being could be explained by the fact that a population often living in poverty is prone to a difficulty of social life in general including undernourishment that would expose it to malnutrition. Among the consequences of malnutrition, we have a decrease of immunity and therefore susceptibility to catch infections including bacteria [15, 44, 45].

Regarding the type of health zones, the most affected in our study were the urban areas, e.g., Kinshasa, Lubumbashi and Kisangani. This could be explained by the poverty that one encounters in urban settlements due to a large demography of multifactorial origin including the rural exodus and immigration of populations from neighboring countries (e.g., Aru, Ariwara, Mahagi), which can be healthy carriers of bacteria, and which fuels bacterial importation [2, 46]. This is more evident in some studies carried out in the countries of the meningitis belt, where the poverty and the incidence of meningitis have been correlated, for example with epidemic outbreaks after large mass gathering pilgrimages [47, 48]. Therefore, it will be necessary to conduct more detailed investigations for the districts that are the more influenced by the introduction of the disease from surrounding countries in order to reinforce this hypothesis.

Surveillance data for meningitis in the DRC have some limitations that should not bias our analyses. This is confirmed by a recent publication on the evaluation of IDSR in Africa with more than 85% data completeness and implementation of IDSR in 50 to 85% of structures at the peripheral level in 2017 [49]. First, these data correspond to suspected cases and a low proportion is actually tested for biological confirmation. Similarly to the studies performed in the African meningitis belt, the surveillance data do not reflect the exact number of meningitis cases with potential under estimations of cases, mainly during epidemics. Meanwhile, under-reporting of cases can occur because of the poor training of care providers and sometimes due to a lack of data transmission tools to the hierarchy. Biological confirmations of routine suspect cases are almost non-existent especially in rural areas. The biological confirmation protocol of the cases defined by the WHO requires a respect of norms and a technical platform not always accessible in the structures of care (reagents, materials for cerebrospinal fluid transport, culture and others). Difficulties related to the collection of cerebrospinal fluid (CSF) are transport and the use of certain methods of laboratory diagnosis (e.g., microscopic exam, culture, biochemical analyses useful for diagnosis, soluble antigens search, Gene amplification…). In DRC, data quality control is performed internally within the Directorate for Disease Control at a quarterly pace. And this direction is also assessed by the DRC General Secretariat for Health at a quarterly rate and during these evaluations; special attention is paid to the quality of surveillance data. Also, annually, the Directorate for Disease Control in particular and the Ministry of Health in general are subject to external quality control of surveillance data (by South Africa national laboratory and CDC Atlanta for Laboratory Data). Another limitation is the population data, which correspond to estimates based on a 1984 census with application of a calculated annual growth rate of 1.03%. That is the only one available and accepted by all actors in this country. The biological confirmation data are useful for determining the case confirmation rate that remains low for several reasons, including the low availability of diagnostic materials and inputs in provinces, but also the transport of CSF samples (transport medium Trans-isolate from cerebrospinal fluid) [28].

### Conclusion

Our results show for the first time that the incidence of meningitis cases and attack rates in the DRC are strongly associated with some key-environmental drivers such as altitude, latitude, longitude, mean and maximum temperature, vegetation biomes and climate categories. Obviously also, important demographic parameters like population size and density in the different health districts and the index of national well-being developed for DRC are important to consider in the development of meningitis epidemic outbreaks over the country. However, when these demographic and socio-economic factors are considered in our models, important biogeographical patterns of meningitis are also revealed.

Our study demonstrates that all other parameters kept constant, an urban health zone located at high latitude and longitude eastwards, located at low-altitude like in valley ecosystems predominantly covered by savannah-type landscape, with a humid tropical climate and a mean range temperature, with a population with a low IEW constitutes the perfect conditions for the development of meningitis. Interestingly, some of the explanatory parameters in the development of meningitis disease in DRC, i.e., bioclimatic factors, are highly sensitive to global environmental changes, and particularly climate change. We strongly recommend to better survey meningitis in the DRC and more generally in countries located outside the meningitis belt because several epidemiological patterns could be altered by global environmental changes, and particularly within the current context of global warming.

**Ethics approval and consent to participate:** This research was not considered human subjects research. Therefore, consultation of an ethics committee and consent to participate is not required.

## Supporting information

S1 Data Data base that allowed modeling. File in.xlsx extension.
(XLSX)
